# Physiological and metabolic responses of *Zymomonas mobilis* to lignocellulosic hydrolysate

**DOI:** 10.1128/spectrum.00610-25

**Published:** 2025-09-17

**Authors:** Julio Rivera Vazquez, Edna Trujillo, Zach Wenger, Michael Botts, Joshua J. Coon, Daniel Amador-Noguez

**Affiliations:** 1 DOE Great Lakes Bioenergy Research Center, University of Wisconsin-Madison 5228, Madison, Wisconsin, USA; 2 Department of Bacteriology, University of Wisconsin-Madison 5228, Madison, Wisconsin, USA; 3 Genome Center of Wisconsin, Madison, Wisconsin, USA; 4 Morgridge Institute for Research 145254, Madison, Wisconsin, USA; 5 Department of Chemistry, University of Wisconsin-Madison 5228, Madison, Wisconsin, USA; 6 Department of Biomolecular Chemistry, University of Wisconsin-Madison 5228, Madison, Wisconsin, USA; Rutgers The State University of New Jersey, Piscataway, New Jersey, USA

**Keywords:** stress response, lignocellulose, lipidomics, proteomics, hydrolysates, biofuels, *Zymomonas mobilis*, metabolism

## Abstract

*Zymomonas mobilis* is a promising biocatalyst for the sustainable conversion of lignocellulosic sugars into biofuels and bioproducts, yet its response to lignocellulosic hydrolysates remains poorly understood. Here, we investigate the physiological response of *Z. mobilis* to ammonia fiber expansion (AFEX)-pretreated switchgrass hydrolysate using a systems-level approach integrating LC–MS/MS-based lipidomics and shotgun proteomics. Growth on hydrolysate induced substantial shifts in fatty acid and membrane phospholipid composition, alongside broad proteomic remodeling. Notably, *Z. mobilis* exhibited a stress response characterized by the upregulation of heat shock proteins and efflux transporters and the downregulation of cell motility proteins. Unexpectedly, hydrolysate exposure also led to a robust upregulation of the Entner–Doudoroff pathway, the ethanol fermentation pathway, and other central carbon metabolism enzymes, indicating a substantial cellular investment potentially driven by additional nutrient availability in hydrolysate. These findings provide new insights into the metabolic adaptations of *Z. mobilis* to lignocellulosic hydrolysates, informing strategies to enhance its biofuel production capabilities.

**IMPORTANCE:**

Biomass pretreatment processes release fermentable sugars from lignocellulosic biomass, but they also generate inhibitors that can impact microbial metabolism. This study provides a systems-level evaluation of how *Zymomonas mobilis* responds to hydrolysate stress, revealing distinct physiological and lipid membrane remodeling responses. While some stress responses overlap with those induced by ethanol and isobutanol toxicity, both valuable biofuels, hydrolysate exposure elicits unique metabolic shifts. These findings offer valuable insights for engineering *Z. mobilis* strains with improved tolerance and performance for efficient bioconversion of lignocellulosic hydrolysates into biofuels and bioproducts.

## INTRODUCTION

The microbial conversion of plant biomass into fuels and chemicals offers a sustainable alternative to fossil-based resources. However, efficient biofuel production requires engineering microorganisms that not only achieve high product yields and titers but also exhibit robustness against stresses encountered during industrial fermentation processes. These stresses include the toxicity of target products at high concentrations and inhibitory compounds present in lignocellulosic hydrolysates produced during biomass pretreatment.

*Zymomonas mobilis*, an aerotolerant anaerobic α-proteobacterium, is a promising industrial organism due to its high catabolic rate, low biomass yield, broad pH tolerance, and increasingly versatile set of genetic engineering tools (1–4). It naturally converts up to 96% of glucose into ethanol and can be engineered to produce other valuable bioproducts, such as isobutanol and isoprenoids (5–11). However, unlike cellulolytic bacteria, *Z. mobilis* lacks the ability to degrade lignocellulosic biomass, necessitating physicochemical pretreatment to break down lignin and hydrolyze cellulose and hemicellulose into fermentable sugars such as glucose and xylose. While essential, these pretreatment processes often generate inhibitory byproducts that can hinder microbial growth and fermentation efficiency (12).

Previous work from our group examined how exposure to ethanol and isobutanol affects the membrane lipid composition and overall physiology of *Z. mobilis* (13). Our findings revealed distinct responses to these stressors: ethanol exposure leads to increased cyclopropane fatty acid (CFA) content and upregulation of CFA synthase, whereas isobutanol exposure decreases CFA content despite robust CFA synthase expression. Isobutanol also triggers a broad stress response, characterized by the upregulation of heat shock proteins, efflux transporters, and DNA repair systems, along with the downregulation of cell motility proteins. While ethanol induces similar changes, its effects were less pronounced. Additionally, isobutanol causes widespread metabolic dysregulation, evident from increased nucleotide degradation intermediates and depletion of biosynthetic and glycolytic intermediates.

Despite growing interest in *Z. mobilis* as a biocatalyst, its physiological response to lignocellulosic hydrolysates remains poorly understood. A deeper understanding of this response would help guide metabolic engineering strategies aimed at improving strain robustness and performance under industrially relevant conditions. Here, we employ a systems-level approach integrating LC–MS/MS-based lipidomics and proteomics to investigate *Z. mobilis* response to ammonia fiber expansion (AFEX)-pretreated switchgrass hydrolysate (ASGH). Our findings reveal that growth on hydrolysate induces substantial shifts in lipid membrane composition and extensive proteomic remodeling, revealing a complex stress response and reprogramming of central carbon metabolism. These insights can inform the engineering of *Z. mobilis* for enhanced biofuel and bioproduct production from lignocellulosic hydrolysates.

## RESULTS AND DISCUSSION

### Experimental design

*Z. mobilis* (ATCC 31821) was inoculated anaerobically at an initial OD₆₀₀ of 0.045 in minimal media or 7% glucan-loading AFEX-pretreated switchgrass hydrolysate (7% ASGH), diluted to 25%, 50%, and 100% (undiluted) (12). AFEX pretreatment releases fermentable sugars while minimizing inhibitor formation compared to acidic pretreatment methods. It improves biomass digestibility without extensive washing, retains essential nutrients such as amino acids, and operates under mild conditions, thereby reducing costs and environmental impact. Table S1 summarizes the concentrations of all measurable components in the AFEX-pretreated hydrolysate used in this study. These advantages make AFEX pretreatment an efficient and scalable option for biofuel and bioproduct production (12). Cultures were grown until reaching an OD₆₀₀ of 0.5, after which samples were collected for lipidomics, proteomics, and microscopy analyses (see Materials and Methods).

### Hydrolysate impacts *Z. mobilis* morphology

**Fig 1:**
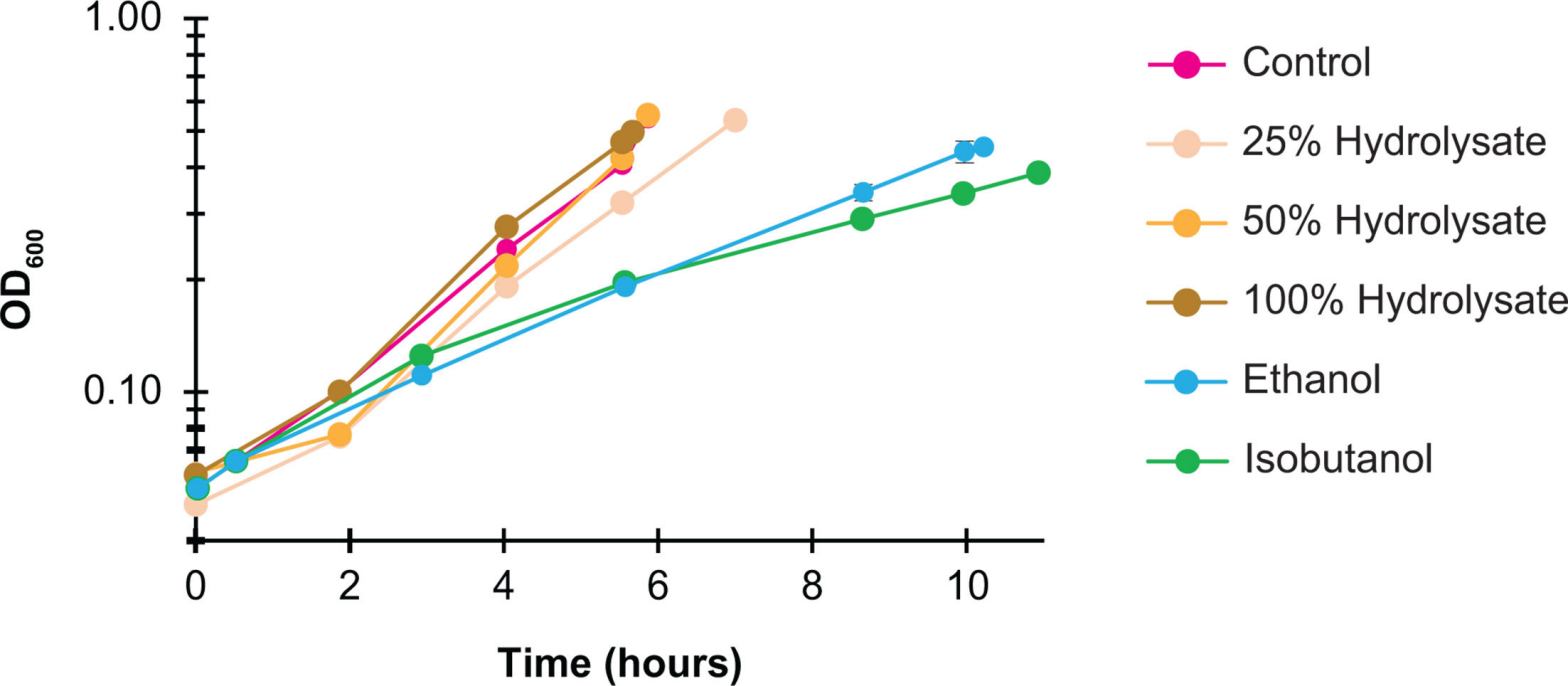
Anaerobic growth of *Z. mobilis* ZM4 in minimal media (control), ASGH diluted to 25%, 50%, and 100% (undiluted), or minimal media supplemented with ethanol (0.80 M) or isobutanol (0.15 M). Each data point represents the average of three to four biological replicates, with error bars indicating standard deviation.

*Z. mobilis* exhibited an average doubling time of 1.7 hours in minimal media. Surprisingly, growth in switchgrass hydrolysate did not negatively affect the growth rate (Fig. 1). Doubling times for cells grown in hydrolysate were 1.8, 1.4, and 1.7 hours for 25%, 50%, and 100% hydrolysate concentrations, respectively (Table S2A). This contrasts with the effects of ethanol or isobutanol exposure, both of which significantly decrease *Z. mobilis* growth rate (Fig. 1).

**Fig 2:**
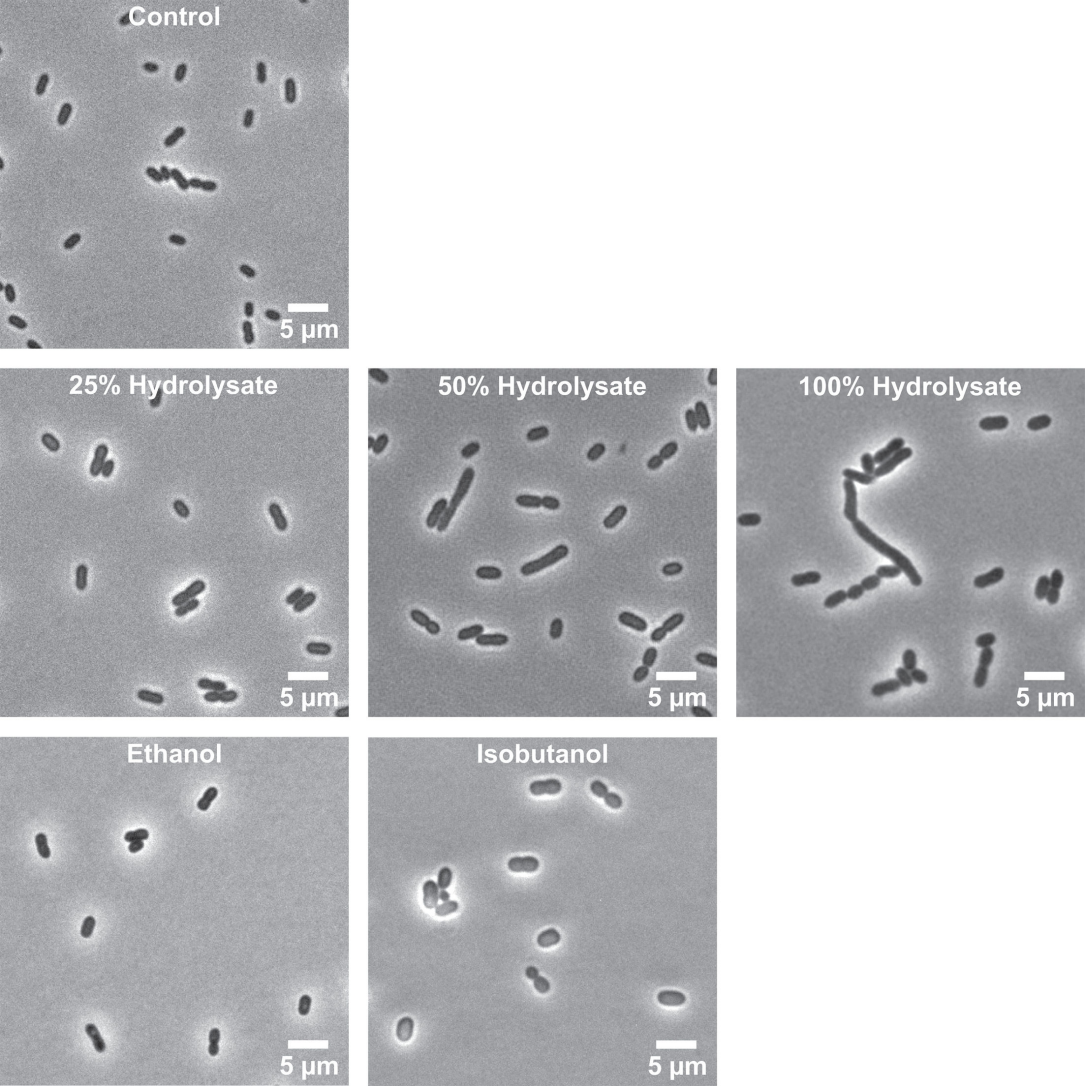
Bright-field microscopy evaluation of cell morphological changes when *Z. mobilis* was grown anaerobically in minimal media, ASGH diluted to 25%, 50%, and 100% (undiluted), or minimal media supplemented with ethanol (0.80 M) or isobutanol (0.15 M).

Microscopy analysis revealed notable morphological changes when *Z. mobilis* was cultured in hydrolysate. Cells became larger and more elongated, particularly at 50% and 100% hydrolysate concentrations (Fig. 2). In minimal media, the average cross-sectional area of *Z. mobilis* cells was 2.7 µm², but in 100% hydrolysate, it increased to an average of 6.2 µm², with individual cell sizes ranging from 3 µm² to 29.7 µm². Similarly, the average cell perimeter increased from 6.4 µm in control cells to 11.3 µm in 100% hydrolysate (Table S3).

The underlying cause of these morphological changes remains unclear. One possibility is that elongation reflects stress-induced disruption of cell division, as has been observed in other bacteria—such as *E. coli*—in response to DNA damage, envelope stress, or metabolic imbalance (14). In the context of lignocellulosic hydrolysate, inhibitors such as phenolics or aldehydes could interfere with peptidoglycan synthesis or septation, leading to filamentation. Alternatively, elongation may represent a transient adaptive response that increases surface-area-to-volume ratio, potentially enhancing nutrient uptake, efflux of toxic compounds, or membrane remodeling. Further research is needed to determine whether this phenotype contributes to hydrolysate tolerance or reflects impaired cell physiology.

### Alterations in fatty acid composition during growth on hydrolysate

Membrane phospholipids in *Z. mobilis* primarily contain six fatty acids: palmitoleic (16:1), myristoleic (14:1), vaccenic (18:1), myristic (14:0), palmitic (16:0), and the cyclopropane fatty acid cis-11,12-methyleneoctadecanoic acid (19:Cyclo). Among these, vaccenic acid (18:1) and palmitic acid (16:0) are the most abundant, comprising ~80% and ~11% of total membrane fatty acids, respectively, whereas the cyclopropane fatty acid (19:Cyclo) accounts for ~2% of the membrane composition (15).

**Fig 3:**
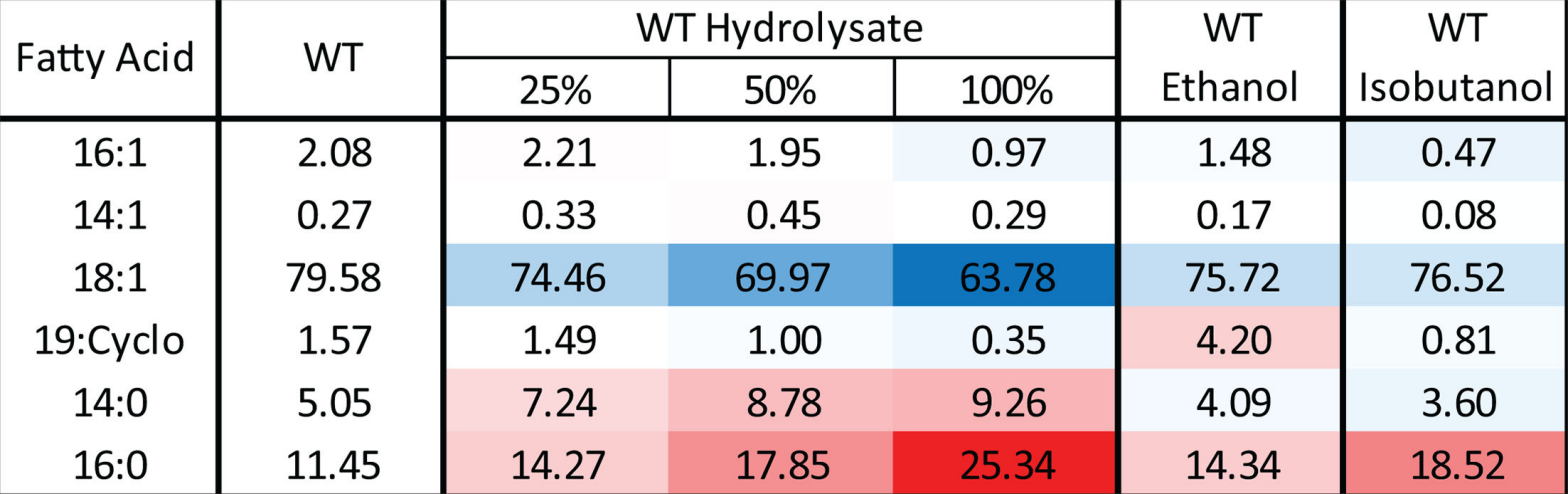
Membrane fatty acid composition of *Z. mobilis* grown anaerobically in minimal media (control), ASGH diluted to 25%, 50%, and 100% (undiluted), or minimal media supplemented with ethanol (0.80 M) or isobutanol (0.15 M). The values indicate the mean weight percentages of fatty acids from three to four independent biological replicates, normalized to total membrane fatty acid content. Cell colors indicate the percent difference relative to the wild-type control, with blue denoting a decrease and red an increase, ranging from −15.8% to +13.9%. Fatty acid names indicate the number of carbon atoms and unsaturations; 19:Cyclo corresponds to cis-10,11-methyleneoctadecanoic acid.

Growth in hydrolysate led to significant changes in membrane fatty acid composition. Most notably, palmitic acid (16:0) content increased significantly from 11.5% under basal conditions to 25.3% in 100% hydrolysate, while vaccenic acid (18:1) decreased from 79.6% to 63.8% (Fig. 3; Table S4A). Similar changes occur during ethanol and isobutanol exposure, although they are significantly more pronounced in hydrolysate. Additionally, myristic acid (14:0) levels nearly doubled, rising from 5.1% to 9.3% in 100% hydrolysate, contrasting with its decline during ethanol and isobutanol exposure.

The cyclopropane fatty acid (19:Cyclo), which constituted 1.6% of membrane fatty acids under basal conditions, decreased more than fourfold to 0.35% in 100% hydrolysate. This decline contrasts with its ethanol-induced increase but aligns with the decrease observed during isobutanol exposure, although the effect is more pronounced in hydrolysate.

### Alterations in phospholipid composition during growth on hydrolysate

The major membrane lipid classes in *Z. mobilis* are phosphatidylethanolamine (PE), phosphatidylglycerol (PG), phosphatidylcholine (PC), and cardiolipin (CL). Among these, PE and PG are the most abundant, comprising approximately 58% and 24% of total phospholipids, respectively, while PC accounts for ~15%, and CL is the least abundant at 3% (13).

**Fig 4:**
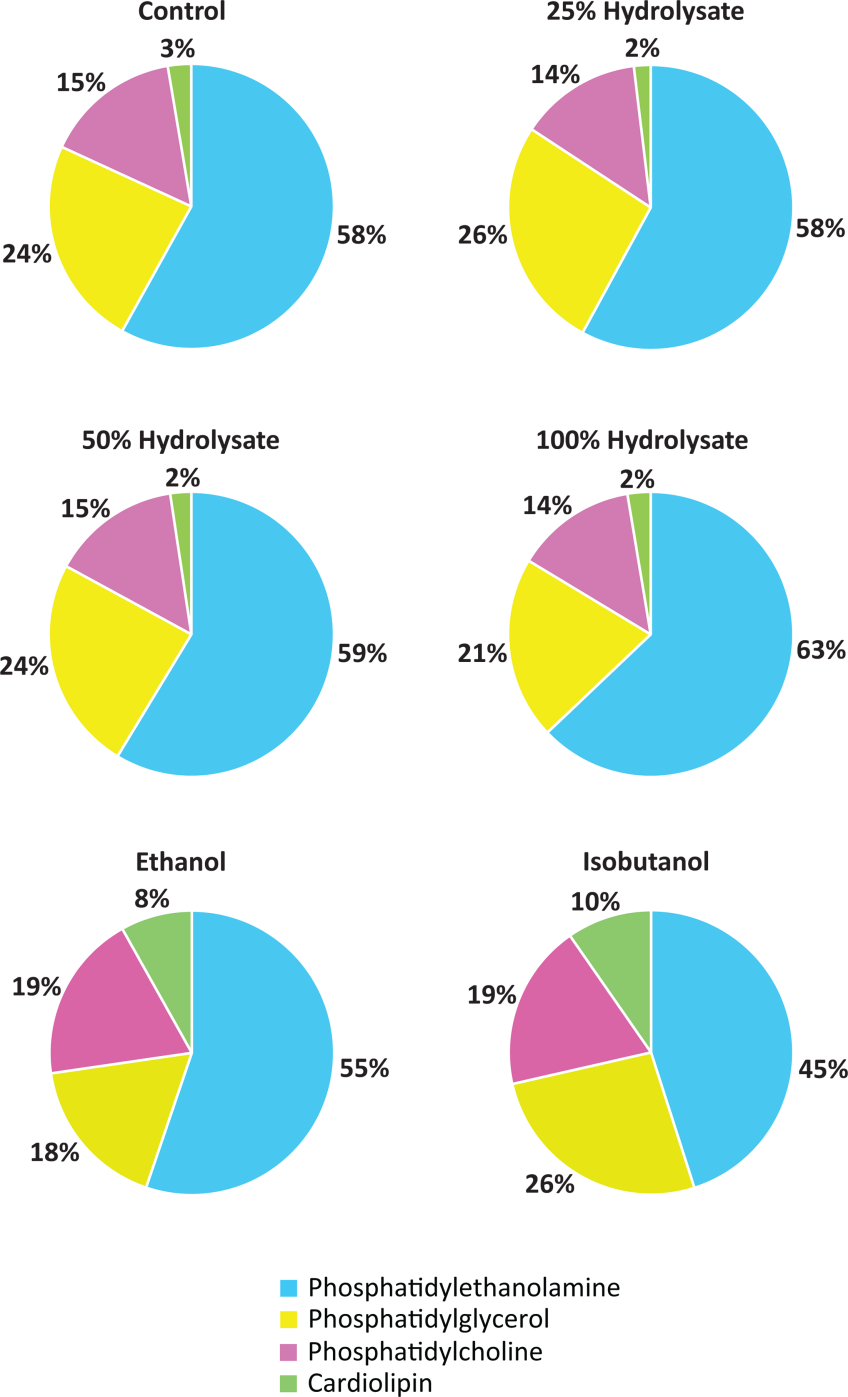
Membrane phospholipid composition of *Z. mobilis* grown anaerobically in minimal media (control), ASGH diluted to 25%, 50%, and 100% (undiluted), or minimal media supplemented with ethanol (0.80 M) or isobutanol (0.15 M). Values represent the percentage of each phospholipid class, calculated as the sum of all individual phospholipids within that class relative to the total measured membrane phospholipid content. Data are averaged from three to four independent biological replicates. Detailed individual phospholipid compositions are provided in Fig. 5.

Growth in hydrolysate resulted in only minor changes to the relative proportions of these lipid classes (Fig. 4; Table S4C). PE content increased slightly from 58.1% under basal conditions to 62.9% in 100% hydrolysate, while PC and PG levels decreased modestly from 15.4% to 13.7% and 23.8% to 20.7%, respectively. These alterations contrast with ethanol and isobutanol exposure, both of which induce more pronounced shifts in overall lipid class composition.

**Fig 5:**
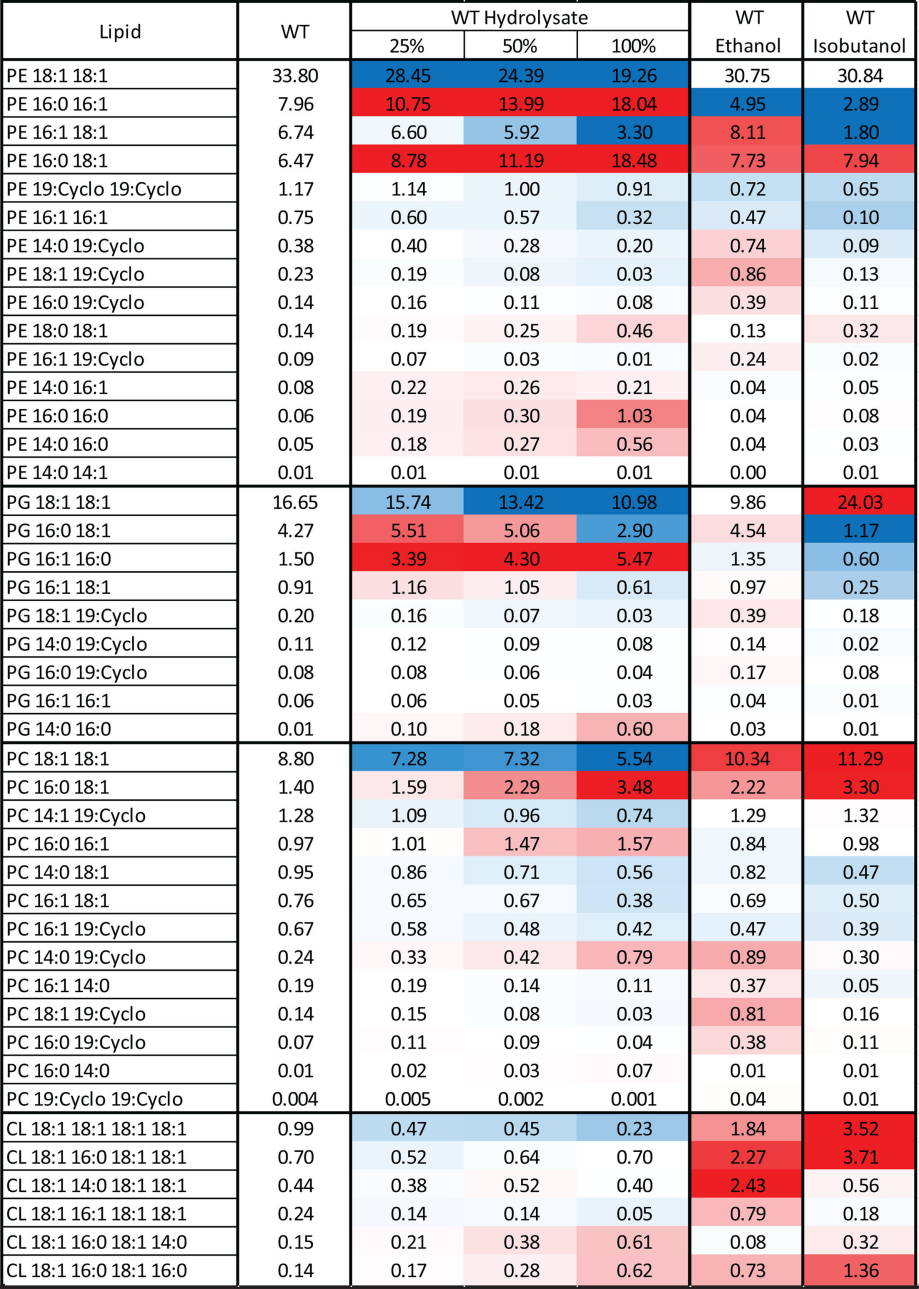
Membrane phospholipid composition of *Z. mobilis* grown anaerobically in minimal media (control), ASGH diluted to 25%, 50%, and 100% (undiluted), or minimal media supplemented with ethanol (0.80 M) or isobutanol (0.15 M). Values represent the average weight percentages of phospholipids from three to four independent biological replicates, relative to the total measured membrane phospholipid content. Cell colors indicate the percent difference compared to the wild-type control, with blue signifying a decrease and red an increase, ranging from −14.5% to +12%. Each phospholipid name specifies the lipid class (PE, PG, PC, or CL) and its component fatty acid tails. Abbreviations: PE, phosphatidylethanolamine; PG, phosphatidylglycerol; PC, phosphatidylcholine; and CL, cardiolipin (CL). 19:Cyclo corresponds to cis-10,11-methyleneoctadecanoic acid.

Despite these minor changes in overall lipid class distribution, we observed significant shifts in the abundance of specific phospholipids (Fig. 5; Table S4B). Under basal conditions, vaccenic acid (18:1)-containing phospholipids dominate, with PE 18:1/18:1 being the most abundant—representing 33.8% of total phospholipids—followed by PG 18:1/18:1 at 16.6% and PC 18:1/18:1 at 8.8%. Together, these three phospholipids account for nearly 60% of the total phospholipid pool.

Consistent with the reduction in vaccenic acid (18:1) content during hydrolysate growth, phospholipids containing two vaccenic acid tails showed large decreases (Fig. 5). Specifically, PE 18:1/18:1 levels dropped sharply from 33.8% to 19.3% in 100% hydrolysate. While ethanol and isobutanol exposure also resulted in decreased levels of this phospholipid, the effect was much less pronounced: ~30.8% in both cases. Similarly, PG 18:1/18:1 decreased from 16.7% to 11.0%, and PC 18:1/18:1 declined from 8.8% to 5.5%. These decreases contrast with isobutanol exposure, where both of these phospholipids increased significantly.

In contrast, phospholipids containing palmitic acid (16:0) increased substantially during growth in hydrolysate (Fig. 5), mirroring the more than twofold rise in 16:0 levels observed in the total fatty acid pool (Fig. 3). In 100% hydrolysate, notable increases included PE 16:0/16:1 (from 8.0% to 18.0%), PE 16:0/18:1 (from 6.5% to 18.5%), PG 16:1/16:0 (from 1.5% to 5.5%), and PC 16:0/18:1 (from 1.4% to 3.5%).

Finally, in agreement with the decline in cyclopropane fatty acid (19:Cyclo) content, nearly all phospholipids containing this fatty acid decreased in 100% hydrolysate. Overall, the changes in the individual phospholipid levels during growth in hydrolysate were substantial and distinct from those observed during both isobutanol and ethanol exposure.

### Proteome remodeling during hydrolysate growth

**Fig 6:**
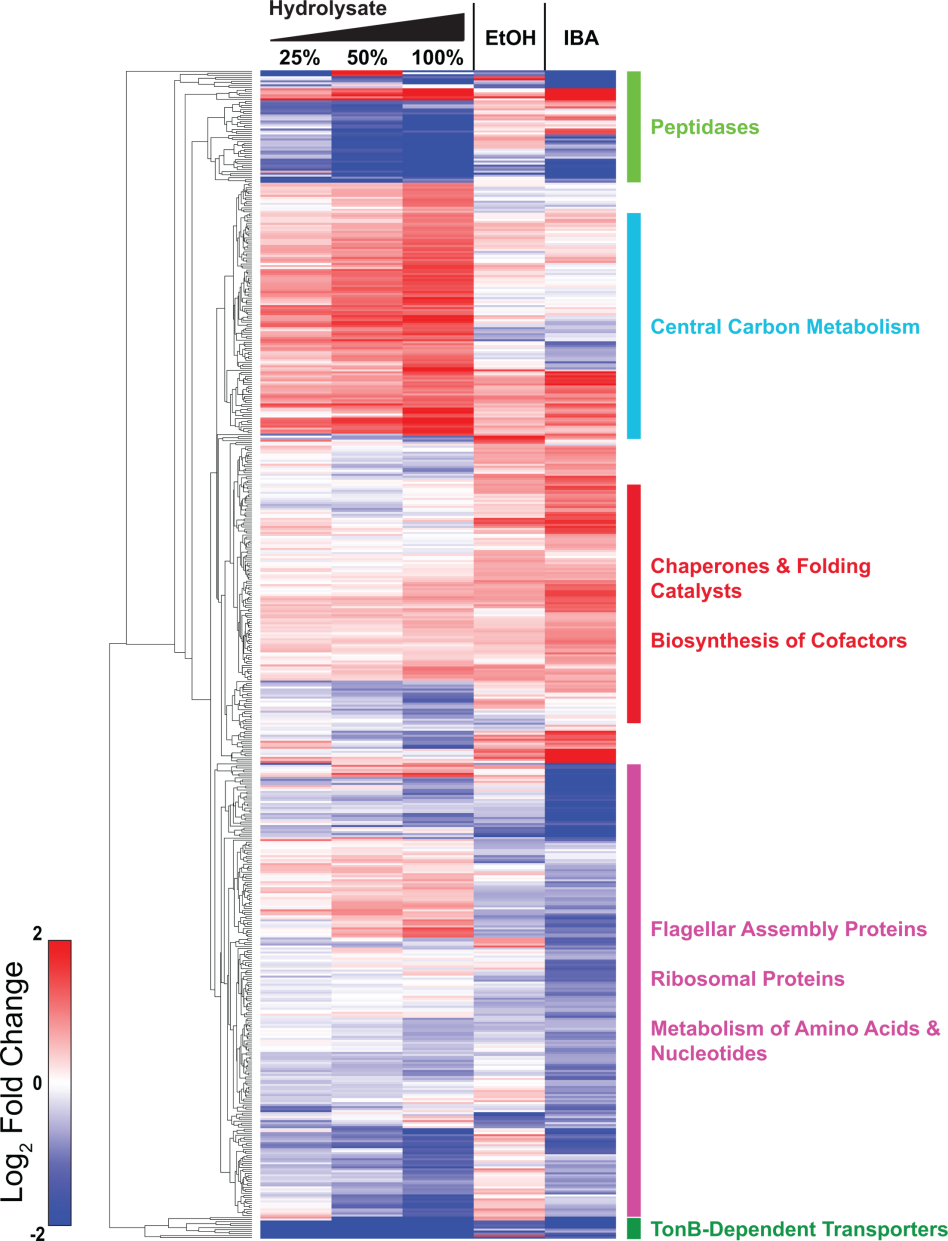
Relative changes in protein abundance compared to controls grown in minimal media during growth on ASGH or minimal media supplemented with ethanol (0.80 M) or isobutanol (0.15 M). Only proteins with a greater than twofold change (in either direction) and an FDR-adjusted *P*-value < 0.05 in at least one condition are shown. Each row represents a single protein, with changes displayed as log₂-fold differences relative to unexposed controls. Red indicates increased protein levels, while blue indicates decreased protein levels. Data represent the average of three independent biological replicates. Protein names, gene IDs, and the complete proteomics data set are provided in Table S5A.

We performed LC–MS/MS-based shotgun proteomic analysis to investigate the physiological responses of *Z. mobilis* during growth on hydrolysate. Of the 1,890 protein-coding genes in the *Z. mobilis* ZM4 genome (4, 16), we quantified the relative abundances of 1,107 proteins. Among these, 245 exhibited significant changes in abundance (FDR-adjusted *P* < 0.05), showing more than a twofold increase or decrease in response to hydrolysate growth (Fig. 6; Table S5). Growth on hydrolysate led to widespread alterations across multiple cellular processes, including flagellar assembly, peptidase activity, outer membrane receptor function, carbohydrate metabolism, chaperone activity, ribosomal protein levels, and nucleotide and amino acid metabolism (Fig. 6). In the following sections, we summarize a subset of the most significant alterations.

### Upregulation of heat shock proteins and efflux transporters during growth on hydrolysate parallels that of ethanol and isobutanol exposure

**Fig 7:**
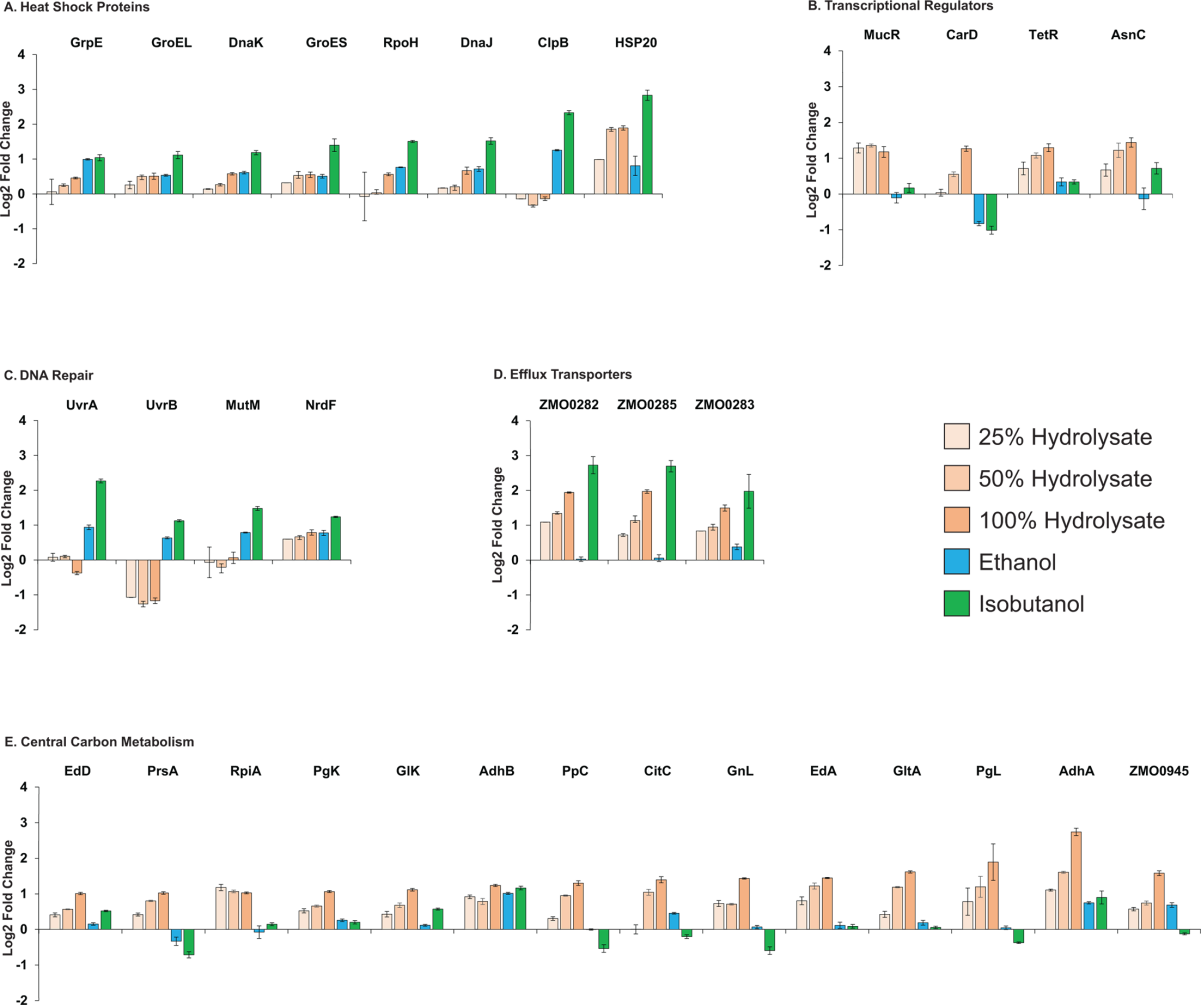
Changes in protein abundance across selected upregulated functional categories during growth on ASGH or minimal media supplemented with ethanol (0.80 M) or isobutanol (0.15 M). Categories include: (**A**) heat shock proteins, (**B**) transcriptional regulators, (**C**) DNA repair, (**D**) efflux transporters, and (**E**) central carbon metabolism. Protein names, gene IDs, and the complete proteomics data set are provided in Table S5. Protein level changes are shown as log₂-fold differences relative to unexposed controls. Data represent the average of three independent biological replicates. Error bars indicate standard deviation.

Heat shock proteins (HSPs) function as molecular chaperones, facilitating protein folding, preventing aggregation, and maintaining proteostasis through refolding or degradation (17, 18). Their expression is triggered by stressors, such as heat, oxidative damage, and cytotoxic compounds (19, 20). During growth on hydrolysate, many HSPs were significantly upregulated, including RpoH (ZMO0749), heat shock protein 20 (ZMO0989), GrpE (ZMO0016), GroES (ZMO1928), DnaK (ZMO0660), DnaJ (ZMO1690), and GroEL (ZMO1929) (Fig. 7). This concerted upregulation of HSPs is similar to that of ethanol and isobutanol exposure, with one notable difference: ClpB, which was strongly upregulated by ethanol and isobutanol, was slightly downregulated during hydrolysate growth. The increased HSP levels may reflect a cellular response to heightened protein turnover resulting from protein damage caused by lignocellulosic-derived inhibitors.

Correlating with the upregulation of HSPs, isobutanol exposure induces protein aggregation in *Z. mobilis* (13). Since hydrolysate exposure triggers a similar, though less pronounced, upregulation of HSPs, we tested whether it also induces protein aggregation. To investigate this, we grew a *Z. mobilis* strain expressing green fluorescent protein (GFP) in hydrolysate and examined protein aggregation using fluorescence microscopy. Unlike isobutanol exposure, hydrolysate growth did not cause widespread protein denaturation and aggregation, as evidenced by the absence of GFP aggregates (Fig. S1).

Efflux transporters play a vital role in bacterial adaptation to adverse environments, actively removing harmful compounds from the cell (21–24). During growth on hydrolysate, we observed a large increase in the abundance of efflux transporters, particularly ZMO0282, ZMO0283, and ZMO0285, with their expression rising progressively as hydrolysate concentrations increased (Fig. 7). It is plausible that *Z. mobilis* upregulates these transporters to expel potentially harmful lignocellulosic-derived inhibitors present in hydrolysate. This response was similar to that previously observed during isobutanol exposure.

### UvrABC repair system levels are less affected by hydrolysate exposure compared to alcohol stress

The UvrABC repair system plays a vital role in detecting and repairing DNA damage. This process begins with a UvrA-UvrB complex scanning the DNA for abnormalities. Upon identifying damage, a UvrB monomer binds to the DNA, using ATP to insert a beta-hairpin between the strands, enabling lesion recognition (25, 26). Ethanol and isobutanol exposure strongly induces UvrABC system proteins, with both UvrA (ZMO1588) and UvrB (ZMO0362) upregulated under solvent stress (13). Additionally, the DNA repair proteins MutM (ZMO1187) and NrdF (ZMO0443) were also elevated. In contrast, hydrolysate exposure triggered a distinct response: NrdF was the only protein upregulated, while UvrB and MutM remained unchanged, and UvrA was downregulated (Fig. 7). These findings suggest that hydrolysate induces less DNA damage than either ethanol or isobutanol.

### Alterations to transcriptional regulators and two-component regulatory systems

During growth on hydrolysate, we observed upregulation of several transcriptional regulators, including MucR (ZMO1412), CarD (ZMO0221), TetR (ZMO0963), and AsnC (ZMO1107) (Fig. 7). This response differed from that seen with isobutanol or ethanol exposure. MucR showed no change under either solvent stress, while CarD was downregulated in both conditions. TetR was upregulated in response to both ethanol and isobutanol, but the increase was more pronounced with hydrolysate. AsnC was only upregulated during isobutanol exposure, with no change under ethanol stress.

**Fig 8:**
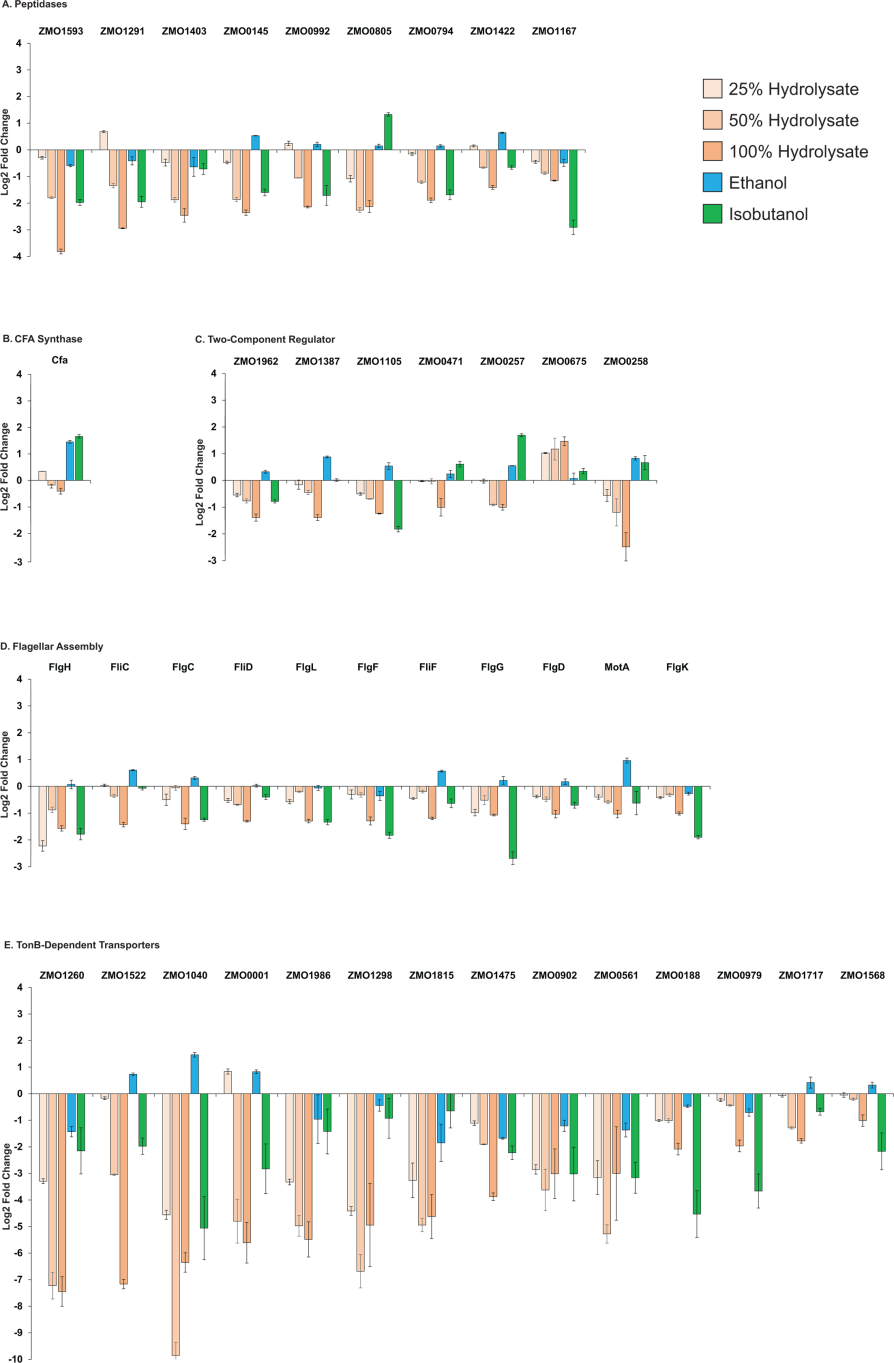
Changes in protein abundance across selected downregulated functional categories during growth on ASGH or minimal media supplemented with ethanol (0.80 M) or isobutanol (0.15 M). Categories include: (**A**) Peptidases, (**B**) two-component regulators, (**C**) cyclopropane fatty acid (CFA) synthase, (**D**) flagellar assembly, and (**E**) TonB-dependent transporters. Protein names, gene IDs, and the complete proteomics data set are provided in Table S5. Protein level changes are shown as log₂-fold differences relative to unexposed controls. Data represent the average of three independent biological replicates. Error bars indicate standard deviation.

Two-component regulatory systems are bacterial signaling pathways in which a membrane-bound sensor kinase detects environmental stimuli and activates a cytoplasmic response regulator to modulate gene expression, allowing cells to respond to changes in environmental conditions (27–29). During growth on hydrolysate, several two-component systems, including ZMO1962, ZMO1387, ZMO1105, ZMO0471, ZMO0257, and ZMO0258, were significantly downregulated, while ZMO0675 was the only member of this class that was upregulated (Fig. 8). The implications of this are unclear. Further research is needed to determine the roles of these regulators in hydrolysate adaptation.

### Downregulation of TonB-dependent transporters

A notable response to hydrolysate growth was the pronounced and coordinated downregulation of TonB-dependent transporters (TBDTs), an effect even more striking than that observed during isobutanol exposure (Fig. 8). TBDTs are outer membrane proteins that mediate the uptake of a broad range of substrates, including ferric chelates (siderophores), vitamin B12, and carbohydrates, from the extracellular environment (30–32). This process is energetically costly, relying on the proton motive force to drive substrate translocation (33, 34). Among all downregulated functional classes, TBDTs showed the most substantial and consistent decrease in abundance.

In some Gram-negative bacteria, TBDTs have been implicated in the uptake of lignin-derived aromatic compounds (35). It is therefore plausible that *Z. mobilis* downregulates these transporters to limit the import of potentially toxic hydrolysate-derived molecules, such as phenolics or aldehydes, which may structurally resemble natural TBDT substrates. In this context, repression of TBDT expression may serve as a protective mechanism to reduce intracellular accumulation of inhibitory compounds.

Alternatively, TBDT downregulation may reflect an energy conservation strategy. Given the high energetic cost of TonB-dependent transport, *Z. mobilis* may downregulate these systems under stress conditions to conserve cellular energy for critical processes, such as protein folding, membrane maintenance, and central metabolism.

A third possibility is that TBDT repression is part of a generalized stress response. In other Gram-negative bacteria, including *E. coli* and *Pseudomonas* spp., global regulators, such as RpoE and CpxR, coordinate the expression of outer membrane proteins in response to envelope stress and redox imbalance (36–38). Although analogous regulatory systems in *Z. mobilis* remain largely uncharacterized, it is plausible that hydrolysate-induced stress activates similar signaling pathways that suppress non-essential or energetically burdensome transporters.

Further research is needed to elucidate the regulatory mechanisms governing TBDT expression in *Z. mobilis* and to determine whether their downregulation confers a direct adaptive advantage during hydrolysate exposure or represents a broader transcriptional response to environmental stress.

### Downregulation of flagellar assembly proteins and peptidases

Flagella are helical filaments that enable bacterial motility, allowing cells to move toward favorable conditions and away from harmful environments (27, 28, 39). During growth on hydrolysate, we observed a coordinated decrease in the levels of several flagellar assembly proteins, including FlgH (ZMO0608), FliC (ZMO0629), FlgC (ZMO0613), FliD (ZMO0651), FlgL (ZMO0604), FlgF (ZMO0610), FliF (ZMO0634), FlgG (ZMO0609), FlgD (ZMO0612), MotA (ZMO0603), and FlgK (ZMO0605) (Fig. 8). This downregulation, similar to that observed during isobutanol exposure, may reflect a cellular strategy to conserve energy by reducing motility-related processes while prioritizing stress defense mechanisms.

Peptidases catalyze the hydrolysis of peptide bonds, breaking proteins and peptides into smaller peptides and amino acids (29, 40). During growth on hydrolysate, several peptidases exhibited reduced expression, including ZMO1593, ZMO1291, ZMO1403, ZMO0145, ZMO0992, ZMO0805, ZMO0794, ZMO1422, and ZMO1167 (Fig. 8). The functional significance of this downregulation remains unclear.

### Downregulation of cyclopropane fatty acid synthesis

Cyclopropane fatty acids (CFAs) help regulate membrane fluidity and permeability, with their rigid cyclopropane rings increasing fatty acid packing density and limiting the diffusion of small organic acids and alcohols across the membrane (41–43). Previously, we found that exposure to ethanol and isobutanol strongly upregulates CFA synthase (13), the enzyme responsible for cyclopropanating unsaturated fatty acids in membrane lipids. However, in contrast to this stress response, growth on hydrolysate led to a decrease in CFA synthase levels (Fig. 8), which correlated with reduced cyclopropane fatty acid (19:Cyclo) content and lower abundance of CFA-containing lipids (Fig. 3 and 5).

**Fig 9:**
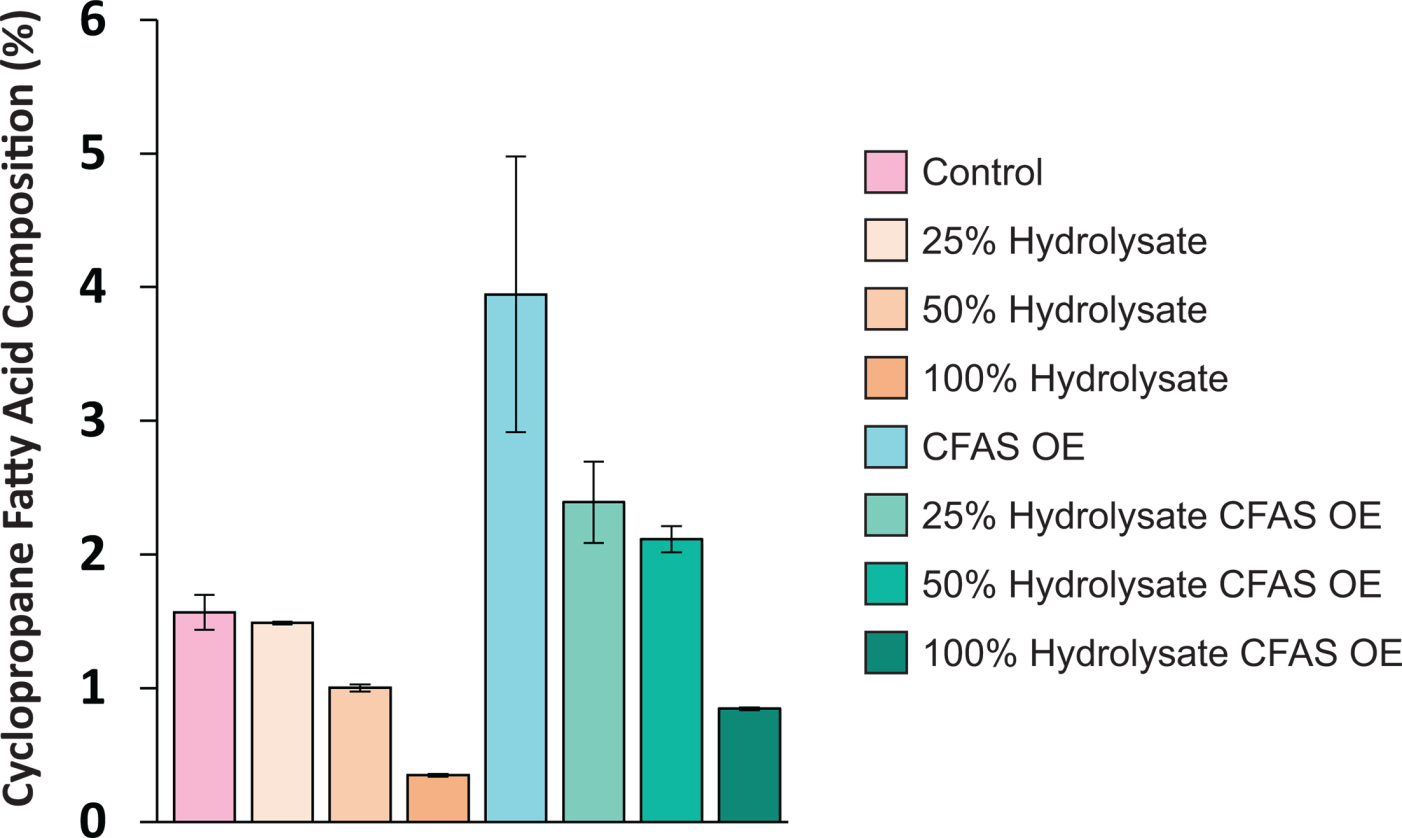
Changes in cyclopropane fatty acid (19:Cyclo) percentage composition in wild-type *Z. mobilis* and a CFA synthase-overexpressing strain grown in minimal medium or ASGH diluted to 25%, 50%, and 100% (undiluted). Data represent the mean of three independent biological replicates. Error bars indicate standard deviation.

To assess whether this decline could be mitigated, we grew a *Z. mobilis* strain overexpressing CFA synthase in hydrolysate. In minimal media, this strain exhibited a fourfold increase in 19:Cyclo fatty acids over wild-type levels upon induction. However, in switchgrass hydrolysate, cyclopropane fatty acid levels still declined as hydrolysate concentrations increased (Fig. 9; Fig. S2; Fig. S4A). Despite this overall decline, the CFA synthase-overexpressing strain maintained higher cyclopropane fatty acid levels than the control under the same conditions. Previous studies in *E. coli* suggest that CFA synthase is degraded by an unidentified heat shock regulon–associated protease (44). A similar protease may be upregulated in *Z. mobilis* during hydrolysate growth, leading to CFA synthase degradation. Further research is needed to test this idea and identify the underlying regulatory mechanisms.

### Upregulation of central carbon metabolism enzymes represents a major protein investment

**Fig 10:**
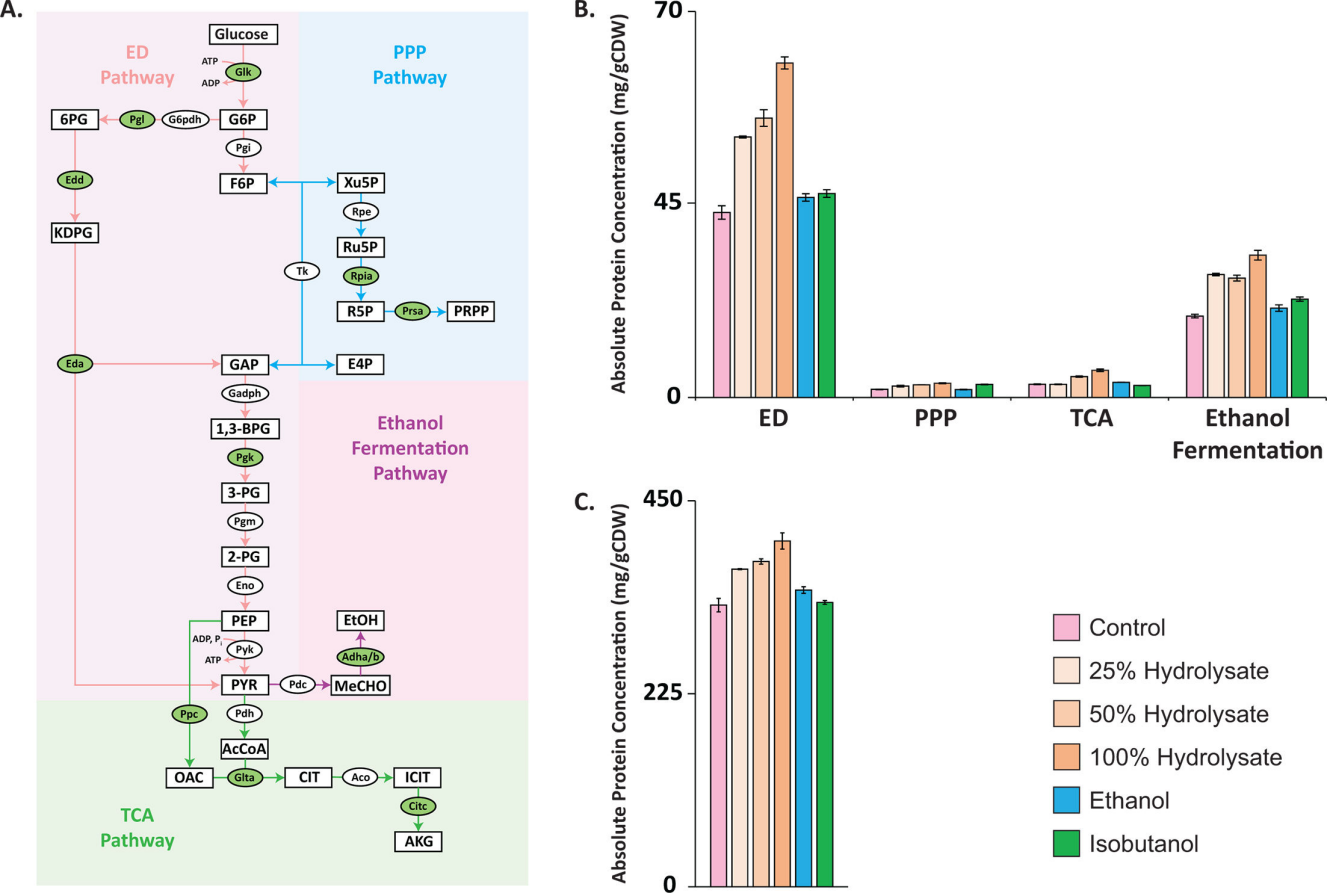
(**A**) Pathways involved in central metabolism of *Z. mobilis*, including the Entner–Doudoroff (ED) pathway, pentose phosphate pathway, citrate cycle, and ethanol fermentation pathway. Metabolites are shown as rectangles, and enzymes as ovals. Enzymes that were upregulated during growth on ASGH are highlighted in green ovals. (**B**) Absolute enzyme concentrations for the ED pathway, pentose phosphate pathway, citrate cycle, and ethanol fermentation pathway, expressed as milligrams per gram of cell dry weight, during growth on ASGH or minimal media supplemented with ethanol (0.80 M) or isobutanol (0.15 M). For the ED pathway, concentrations represent the sum of Glk, Gapdh, Pgk, Pgm, Eno, Pyk, Pdh, Gnl, G6pdh, Pgl, Edd, and Eda. The pentose phosphate pathway concentration is the sum of Pgi, Tk, Rpia, and Prsa. The citrate cycle concentrations represent the sum of Glta, Pdh, Aco, Citc, and Ppc. The ethanol fermentation pathway concentration is the sum of Pdc, Adha, and Adhb. Data represent the average values from three or four biological replicates, with error bars showing standard deviation. (**C**) Absolute concentrations of all measured proteins across the indicated conditions. Metabolite abbreviations: 6-phosphogluconate (6PG), glucose 6-phosphate (G6P), 2-keto-3-deoxy-6-phosphogluconate (KDPG), fructose 6-phosphate (F6P), glyceraldehyde 3-phosphate (GAP), 1,3-bisphosphoglycerate (1,3-BPG), 3-phosphoglycerate (3PG), 2-phosphoglycerate (2PG), phosphoenolpyruvate (PEP), pyruvate (PYR), oxaloacetate (OAA), acetyl CoA (AcCoA), erythrose 4-phosphate, xylulose 5-phosphate (Xu5P), ribulose 5-phosphate (Ru5P), ribose 5-phosphate (R5P), acetaldehyde (MeCHO), ethanol (EtOH), citrate (CIT), isocitrate (ICIT), alpha-ketoglutarate (AKG), and phosphoribosyl pyrophosphate (PRPP).

A striking response of *Z. mobilis* to growth on hydrolysate was the robust upregulation of Entner–Doudoroff (ED) pathway and ethanol fermentation enzymes. Specifically, glucokinase (Glk, ZMO0369), 6-phosphogluconolactonase (PgL, ZMO1478), phosphogluconate dehydratase (Edd, ZMO0368), 2-dehydro-3-deoxy-phosphogluconate aldolase (EdA, ZMO0997), phosphoglycerate kinase (PgK, ZMO0178), alcohol dehydrogenase II (AdhB, ZMO1596), and alcohol dehydrogenase I (AdhA, ZMO1236) all increased their levels by more than twofold (Fig. 7). Given that these enzymes are already among the most abundant proteins in *Z. mobilis* (45), their coordinated upregulation represents a large increase in cellular protein investment (Fig. 10). Additionally, key enzymes in the pentose phosphate pathway and TCA cycle were strongly upregulated. The coordinated increase in central carbon metabolism enzyme levels was unique to hydrolysate growth and absent during ethanol or isobutanol exposure. Reflecting this, total measured protein levels were significantly higher during hydrolysate growth.

This substantial increase in the abundance of glycolytic and ethanol fermentation enzymes was accompanied by a nearly 75% increase in both glucose consumption and ethanol production rates (Table 1; Table S6). Specifically, glucose uptake increased from 43.2 mmol/gCDW/h in minimal media to an average of 73.3 mmol/gCDW/h during hydrolysate growth. Ethanol production rates similarly increased from 77.2 mmol/gCDW/h to an average of 144 mmol/gCDW/h in hydrolysate-grown cultures (Table 1). Notably, ethanol yield—calculated as the percentage of glucose converted to ethanol—increased from approximately 89% in minimal media to an average of 98% in hydrolysate (Table 1). Despite these increases, growth rates remained largely unchanged, rising only slightly from 0.36 h⁻¹ in control conditions to an average of 0.373 h⁻¹ during hydrolysate growth (Table 1; Fig. S3). This indicates that the elevated flux through the ED pathway was directed toward ethanol production rather than biomass synthesis. Together, these results indicate that proteomic reallocation toward central metabolism in hydrolysate-grown *Z. mobilis* supports enhanced carbon flux through the ED pathway and ethanol fermentation without increasing cellular growth.

**TABLE 1:** Specific growth rates, glucose consumption rates, ethanol production rates, and ethanol yields of *Z. mobilis* grown in 25%, 50,% and 100% hydrolysate (7% ASGH)^*a*^

Condition	Specific growth rate (h^−1^)	Glucose consumption (mmol/gCDW/h)	Ethanol production (mmol/gCDW/h)	Ethanol yields (%)
Control	0.36	43.2 ± 2.2	77.2 ± 3.9	89% ± 0.04%
25% hydrolysate	0.37	75.3 ± 0.6	142.0 ± 3.5	94% ± 3%
50% hydrolysate	0.41	71.6 ± 1.5	146.3 ± 11.5	102% ± 6%
100% hydrolysate	0.34	73.0 ± 2.3	143.7 ± 4.1	98% ± 3%

^*a*^ All hydrolysate data are averages of three biological replicates from each condition. Control data was extracted from Reference 46. .

### Conclusion

AFEX-pretreated switchgrass hydrolysate contains a complex mixture of soluble sugars, organic acids, phenolic compounds, and residual lignocellulosic fragments. While the pretreatment process releases fermentable sugars like glucose and xylose, it also generates lignocellulosic-derived inhibitors, such as acetate, furfural, and phenolics, that can impair microbial metabolism (47). Despite the presence of these inhibitory compounds, some at high concentrations, *Z. mobilis* maintained robust growth in hydrolysate. However, as shown in this study, hydrolysate exposure induces substantial changes in lipid membrane composition and proteome expression. These alterations reveal a broad stress response, characterized by the upregulation of heat shock proteins and efflux transporters, alongside the downregulation of cell motility proteins. Some of these responses resemble those induced by isobutanol exposure, though they are generally less pronounced, while others—such as shifts in membrane fatty acid composition—are more prominent during hydrolysate growth.

The ability of *Z. mobilis* to sustain growth despite inhibitory compounds suggests that its defense mechanisms effectively mitigate the effects of lignocellulosic-derived inhibitors. One interesting example is the coordinated downregulation of TonB receptors, which may limit the uptake of toxic lignin-derived molecules. Simultaneously, hydrolysate may provide additional nutrients and alternative carbon sources that help counteract stress. While *Z. mobilis* cannot utilize xylose, it may metabolize other compounds present in hydrolysate, such as amino acids —17 of the 20 proteinogenic amino acids are present in significant amounts (Table S1)— or alternative six-carbon sugars. The strong upregulation of central carbon metabolism enzymes, already among the most abundant proteins in *Z. mobilis*, suggests a substantial investment potentially fueled by increased resource availability. These observations point to a combined effect of stress and nutrient enrichment in shaping the observed physiological response. Dissecting the relative contributions of these factors will require defined media experiments using individual hydrolysate components.

These findings have important implications for industrial biofuel production, where hydrolysate composition can vary and inhibitor levels may be elevated. The ability of *Z. mobilis* to maintain high growth rates and ethanol yields while mounting specific physiological defense responses suggests a notable degree of inherent resilience that may extend to industrial fermentations. However, some adaptations—such as extensive proteome reallocation and altered cell morphology—may involve trade-offs that impact performance under large-scale fermentation conditions. Understanding how these adaptations affect industrial fermentation performance will be essential for guiding future strain engineering and process optimization.

In summary, this study provides a systems-level evaluation of how growth on hydrolysate affects *Z. mobilis* lipid membrane composition and overall physiology. While there is partial overlap with responses to ethanol and isobutanol exposure, hydrolysate elicits a distinct set of metabolic and regulatory changes, most notably the strong upregulation of central carbon metabolism enzymes. These insights provide a foundation for engineering *Z. mobilis* strains with enhanced robustness and productivity for industrial biofuel and bioproduct production from lignocellulosic feedstocks.

## MATERIALS AND METHODS

### Media composition and growth conditions

*Zymomonas mobilis* ZM4 (ATCC 31821) was anaerobically cultured on a rich medium plate at 30°C for three days. A single colony was used to inoculate 6 mL of rich medium, followed by inoculation of 20 mL ZM4 minimal medium with 60 µL of the overnight culture. This culture was grown for 16 hours before being used to seed three 20 mL minimal medium cultures and three additional 20 mL cultures per 7% glucan loading AFEX-pretreated switchgrass hydrolysate (7% ASGH) percentage condition, adjusting to an initial optical density (OD600) of 0.05. Cells were cultivated under different conditions until reaching an OD600 of 0.500 in an anaerobic glove bag. Subsequently, 5 mL of culture was collected and centrifuged at 4,400 rpm for 10 minutes (Allegra X-30R, Beckman Coulter). The resulting cell pellets were flash-frozen and stored at −80°C for lipidomics and proteomics analysis. The anaerobic glove bag maintained an atmosphere of 5% H₂, 5% CO₂, and 90% N₂, with oxygen levels kept below 50 ppm. Rich medium plates were prepared with 10 g/L yeast extract, 2 g/L KH₂PO₄, 18 g/L agar, and 20 g/L glucose, yielding a final pH of 5.72. Minimal medium consisted of 1 g/L K₂HPO₄, 1 g/L KH₂PO₄, 0.5 g/L NaCl, 1 g/L NH₄SO₄, 0.2 g/L MgSO₄·7H₂O, 25 mg/L Na₂MoO₄·2H₂O, 2.5 mg/L FeSO₄·7H₂O, 20 mg/L CaCl₂·2H₂O, 1 mg/L calcium pantothenate, and 20 g/L glucose, with a final pH of 6.45. For hydrolysate conditions, cultures were grown in either 100% hydrolysate or hydrolysate diluted with water to 25% or 50%. Table S1 summarizes the concentrations of all measurable components in the AFEX-pretreated hydrolysate used in this study.

### Lipid extractions

As performed in (13), the frozen pellets were thoroughly washed with 500 µL of water and then pelleted within a 2 mL glass vial (Thomas Fisher, 1234R80). Subsequently, 300 µL of a butanol/methanol solution in a 3:1 (vol/vol) ratio was gently injected using a glass digital analytical syringe (VWR, 97049-424). After this, the solution was strenuously vortexed for 30 seconds and further mixed with a foam tube holder for 10 minutes. In a subsequent step, 150 µL of a hexane/ethyl acetate solution in a 3:1 (vol/vol) ratio was added, vortexed for 30 seconds, and mixed for 5 minutes. This process was performed two times, yielding a total of 300 µL of the hexane/ethyl acetate 3:1 (vol/vol) solution being added. The reaction was completed by the addition of 300 µL of a 1% acetic acid solution. After vortexing for 30 seconds and mixing for 5 minutes, the solution was centrifuged at 4°C for 10 minutes at 4400 rpm. Two hundred microliters of the upper layer were collected for lipid analysis and fatty acid saponification. The collected layer was then dried under a stream of nitrogen gas. The resulting dried lipids were reconstituted in 55 µL of methanol, with 45 µL being used for LC–MS analysis.

### Fatty acid saponification

As described in (13), in a 2 mL glass vial (Thomas Fisher, 1234R80), the dried 200 µL lipid layer was resuspended by adding 1 mL of a methanol/water solution with a ratio of 9:1 (vol/vol) and containing 0.3 M potassium hydroxide. The samples were then incubated in a water bath at 80°C for 1 hour. Afterward, 100 µL of formic acid was added to acidify the solution. To allow phase separation, 900 µL of hexane was added, followed by vortexing for 1 minute and centrifugation at 4,400 rpm for 5 minutes. Subsequently, 600 µL of the uppermost layer was carefully transferred to a new glass vial and dried under nitrogen gas. The dried sample was then reconstituted with 100 µL of a solution of acetonitrile, isopropanol, and water in a 65:30:5 (vol/vol) ratio. For LC–MS analysis, 80 µL of this solution was used.

### Cloning

CFA synthase from *Z. mobilis* was cloned into the pRL814 plasmid, generously provided by Robert Landick (Professor, UW-Madison, Department of Biochemistry, Great Lakes Bioenergy Research Center). This plasmid originated from a pIND4 fragment, which contains the lacIq gene and PT7A1-O34, and a pRH52 fragment, which carries gfp, the pBBR-1 broad-host-range origin of replication, and aadA for spectinomycin resistance. To assemble these constructs, primers were meticulously designed using the NEB Builder software (https://nebuilderv1.neb.com/), enabling the amplification of gene and plasmid backbone fragments from template DNA, whether genomic or plasmid. Each gene segment was equipped with a unique ribosome binding site (RBS) ranging from 20 to 30 base pairs in length, designed using the RBS Library Calculator. These DNA fragments were designed to overlap by 20–30 base pairs and were seamlessly integrated into the pRL814 plasmid. Gibson assembly reactions were performed using NEB “Hi-Fi” reagents and contained approximately 0.015 pmol of total plasmid backbone DNA, with variable gene fragment DNA (ranging from 0.03 pmol to 0.09 pmol) in a final volume of 25 µL. Reactions were incubated for 1 hour at 50°C. Subsequently, *E. coli* DH5α cells were transformed and selectively screened with 100 µg/mL spectinomycin. The identified colonies underwent scrutiny to confirm successful cloning through plasmid extraction and region-specific gene sequencing. The verified constructs were then introduced into either ZM4 triple (ΔhsdSc, Δmrr, and Δcas3) or quadruple (ΔhsdSc, ΔhsdSp, Δmrr, and Δcas3) mutant strains of ZM4 using a defined conjugation protocol. The resulting ZM4 colonies were meticulously screened, and conjugation was validated through PCR and sequencing. Finally, glycerol stocks of these engineered strains were prepared and stored at −80°C.

### LC–MS/MS lipidomics

The LC–MS lipid method was performed using a Vanquish ultra-high-performance liquid chromatography (UHPLC) system (Thermo Scientific) coupled to a hybrid quadrupole-Orbitrap mass spectrometer (Q Exactive; Thermo Scientific). The chromatography was performed on a reverse-phase C18 column (Acquity UPLC CSH) (1.7 µm particle size, 2.1 × 100 mm column). Solvent A was 70% acetonitrile and 30% H2O with 10 mM ammonium acetate and 250 µL acetic acid per liter. Solvent B was 90% isopropanol and 10% acetonitrile with 10 mM ammonium acetate and 250 µL acetic acid per liter. The total run time was 33 minutes. Flow rate was held constant at 0.4 mL/min. The chromatography gradient was as follows: 2% solvent B for 2 minutes; linear increase to 30% B over 3 minutes; linear increase to 85% B for 14 minutes; linear increase to 99% for 1 minute; maintenance at 99% B for 7 minutes; linear decrease back to 2% B over 1 minute; and maintenance at 2% B for 5 minutes. For the general method, eluent from the column was analyzed by MS from the start of the run until 28 minutes, at which time the flow was directed to waste for the remainder of the run. The quantitation of phospholipids was conducted via external calibration using the following standards: cardiolipin 16:0 16:0 16:0 16:0, cardiolipin 18:1 18:1 18:1 18:1, phosphatidylethanolamine 14:0 14:0, phosphatidylethanolamine 16:0 16:0, phosphatidylethanolamine 18:0 18:1, phosphatidylethanolamine 16:0 18:1, phosphatidylethanolamine 18:1 18:1, phosphatidylglycerol 14:0 14:0, phosphatidylglycerol 16:0 16:0, phosphatidylglycerol 18:0 18:0, phosphatidylglycerol 18:1 18:1, phosphatidylcholine 16:0 18:1, and phosphatidylcholine 18:1 18:1. These lipid standards were purchased from Avanti Polar Lipids, Inc. For many of the identified phospholipid species, purified standards are not commercially available. In such cases, the most similar standard, in terms of phospholipid class, chain length, and structure, was used for quantification purposes. The mass spectrometry lipidomics data have been deposited to the MetaboLights repository with the data set identifier MTBLS12808.

### LC–MS analysis of fatty acids

The fatty acid LC–MS method was performed on a Vanquish ultra-high-performance liquid chromatography (UHPLC) system (Thermo Scientific) coupled to a hybrid quadrupole-Orbitrap mass spectrometer (Q Exactive; Thermo Scientific). The chromatography was performed using a reverse-phase C18 column (Acquity UPLC CSH) (1.7 µm particle size, 2.1 × 100 mm column). Solvent A consisted of 60% acetonitrile and 40% H2O with 10 mM ammonium acetate and 250 µL acetic acid per liter. Solvent B consisted of 90% isopropanol and 10% acetonitrile with 10 mM ammonium acetate and 250 µL acetic acid per liter. The total run time was 33 minutes. Flow rate was held constant at 0.4 mL/min. The chromatography gradient was as follows: 2% solvent B for 2 minutes, linear increase to 30% B over 16 minutes, linear increase to 85% B for 5 minutes, linear increase to 99% for 1 minutes, maintenance at 99% B for 3 minutes, linear decrease back to 2% B over 1 minute, maintenance at 2% B for 5 minutes. For the general method, eluent from the column was analyzed by MS from the start of the run until 28 minutes, after which the flow was directed to waste for the remainder of the run. The quantitation of fatty acids was conducted via external calibration using the following standards: 16:1, palmitoleic acid; 14:1, myristoleic acid; 18:1, vaccenic acid; 19:Cyclo, cis-11,12-methyleneoctadecanoic acid; 14:0, myristic acid; and 16:0, palmitic acid. Fatty acid standards were purchased from Avanti Polar Lipids, Inc. and Matreya, LLC. For some of the fatty acids identified, purified standards are not commercially available. In such cases, the most similar standard, in terms of chain length and structure, was used for quantification purposes.

### LC–MS/MS lipidomics data processing

Raw file data sets were processed using MZmine 2 v2.37 (48). The mass detection was used with a retention time of 0.20–26 minutes. The noise level was 1.0E4 for mass detector in centroid. The chromatogram builder was used with a retention time of 0.20–26.00, a minimum time span of 0.05 minutes, a minimum height of 5.0E4, and an m/z tolerance of 0.005 m/z and 10.0 ppm. The chromatogram deconvolution was conducted with the local minimum search algorithm. The chromatographic threshold was 0.02%; the search minimum in retention time (RT) range was 0.05 min, with a minimum relative height of 0.02% and a minimum ratio of peak top/edge ratio of 3. Peak duration range 0.05–1.50 minutes. Isotopologues were grouped using the isotopic peaks grouper algorithm with an m/z tolerance of 0.005 and 10.0 ppm, an RT tolerance of 0.05 min (absolute), and a maximum charge of 2. The representation isotope was set to most intense. A peak alignment step was performed using the join aligner module (m/z tolerance  =  0.005 m/z and 10.0 ppm, weight for m/z  =  20, RT tolerance  =  0.1 absolute min, weight for RT  =  20). A gap-filling module was used with the same RT and m/z range gap filling (m/z tolerance  =  0.005 m/z and 10.0 ppm). The resulting peak list was then filtered using the Peak List Row Filter module, with a minimum peak intensity of 0.75 per row and a minimum of two peaks per isotope pattern. The peak list was then exported to *.csv using the “Export to CSV file” module with the export common elements (row ID, row m/z, row retention time, row identity (all IDs), row comment, and row number of detected peaks) and export data file elements (peak status, peak m/z, peak RT, peak height, peak area, peak charge, and peak FWHM). The peak list was annotated using LipiDex. Data analysis was conducted using the El-MAVEN software (Elucidata), with compound identification based on retention times matched against authenticated pure standards. Fold changes in lipid, fatty acid, and metabolite concentrations under various growth conditions were quantified relative to *Z. mobilis* cultured in minimal medium. Raw lipidomics data for the ethanol and isobutanol conditions, used for comparisons against hydrolysate treatments, were obtained from Rivera-Vazquez et al (13). Signal intensities were subjected to a log2 transformation, followed by two-tailed *t*-tests with equal variance assumptions to calculate corresponding *P* values.

### Protein extraction

During protein extraction, 10 mL of bacterial culture was collected and centrifuged for 2.5 minutes at 4,000 × *g* at 4°C. The supernatant was discarded, and the cell pellets were frozen in liquid nitrogen and stored at −80°C until further analysis. For proteomics analysis, the cell pellets were thawed and lysed by resuspending in 6 M guanidine hydrochloride. The samples went through three cycles of heating to 100°C for 5 minutes and re-equilibration to room temperature for 5 minutes. Total protein concentration was determined using a Pierce bicinchoninic acid (BCA) protein assay kit (Thermo Scientific), and 50–100 µg of protein was used for further processing. Methanol was introduced to achieve a final concentration of 90%, followed by centrifugation at 15,000 × *g* for 5 minutes. Subsequently, the supernatant was removed, and the protein pellets were desiccated for 10 minutes. These pellets were then reconstituted in 200 µL of lysis buffer (comprising 8 M urea, 100 mM Tris [pH 8.0], 10 mM tris(2-carboxyethyl)phosphine (TCEP) hydrochloride, and 40 mM chloroacetamide) to induce denaturation, reduction, and alkylation of proteins. The resuspended proteins were diluted to 1.5 M urea in 100 mM Tris (pH 8.0). Trypsin was added at a ratio of 50:1 sample protein concentration to trypsin and incubated overnight (approximately 12 hours) at room temperature. The trypsinization reaction was terminated by adding 10% trifluoroacetic acid (TFA). Subsequent to protein digestion, each sample underwent desalting using a Strata-X 33 µM polymeric reversed-phase styrene divinylbenzene solid-phase extraction cartridge and was subsequently dried. Prior to LC–MS/MS analysis, the samples were reconstituted in a 0.2% formic acid solution, and peptide concentrations were quantified employing a Pierce quantitative colorimetric peptide assay kit (Thermo Scientific).

### LC–MS/MS proteomics

For every analysis, 2 µg of peptides were loaded onto a 75-μm inner-diameter (i.d.), 30 cm long capillary featuring an embedded electrospray emitter, and were packed into a C18 BEH column with 1.7 µm particle size. The mobile phases were as follows: phase A, 0.2% formic acid; and phase B, 0.2% formic acid–70% acetonitrile. Peptides were eluted using a gradient that transitioned from 0 to 75% B over 42 minutes, followed by a 4-minute wash with 100% B and a 10-minute equilibration in 100% A, resulting in a comprehensive 60-minute gradient. The eluting peptides were analyzed using an Orbitrap Fusion Lumos mass spectrometer (Thermo Scientific). Survey scans were conducted at a resolution of 240,000, with isolation analysis in the range of 300–1350 m/z and an AGC target of 1e6. Data-dependent top-speed (1 s) tandem MS/MS sampling of peptide precursors was activated, with dynamic exclusion set at 10 s for precursors with charge states ranging from 2 to 4. MS/MS sampling was performed using 0.7 Da quadrupole isolation and fragmentation via higher-energy collisional dissociation (HCD) with a collision energy value of 25%. The mass analysis was carried out within the ion trap using the “turbo” scan speed, spanning a mass range of 200–1,200 m/z. The maximum injection time was configured at 11 ms, and the AGC target was set at 20,000. The mass spectrometry proteomics data have been deposited to ProteomeXchange via the PRIDE partner repository with the data set identifier PXD066951.

### Proteomics data analysis

MaxQuant software (version 1.5.8.3) (49) was used to analyze the raw LC–MS files. The spectra were subjected to a search against a target decoy database using the Andromeda search engine. Label-free quantitation and match between runs were enabled. The MS/MS tolerance was set at 0.4 Da, and all other analysis parameters used their default settings. The peptides were organized into protein groups and filtered to meet a 1% false discovery rate (FDR) criteria based on the target-decoy method. The log2-transformed label-free quantitation intensities were used to calculate log2 fold-change values, compared either to *Z. mobilis* overexpressing GFP or to background signals generated randomly within the noise range. Raw proteomics data for the ethanol and isobutanol conditions, used for comparisons against hydrolysate treatments, were obtained from Rivera-Vazquez et al. (13). The absolute protein quantifications for Fig. 10 are calculated using data from (45).

### HPLC metabolomics

For individual samples, acetate, ethanol, formate, glucose, glycerol, lactate, pyruvate, xylose, xylitol, succinate, and cellobiose were separated by high-performance liquid chromatography (HPLC) and analyzed using refractive index detection (RID). HPLC-RID was performed on an Agilent 1260 Infinity system (Agilent Technologies, Palo Alto, CA) equipped with a 300 × 7.8 mm Aminex HPX-87H anion-exchange column (Bio-Rad, Hercules, CA). Samples were diluted to 10% with Milli-Q water, and 5 µL was injected into the HPLC-RID system. Separation was performed isocratically with 0.02 N H2SO4 at a flow rate of 0.5 mL/min (RID flow cell, 50°C; column, 50°C). Standard compounds were diluted in HPLC-grade water using calibration curves ranging from 0.01 to 20 g/L to generate a standard curve. Analyte concentrations were calculated using Waters Empowering Version 3.8.1.

### Microscopy

*Z. mobilis* and a GFP-overexpressing *Z. mobilis* strain were grown anaerobically at 30°C in minimal medium, ASGH diluted to 25%, 50%, and 100% (undiluted), or minimal medium supplemented with ethanol (0.80 M) or isobutanol (0.15 M). When the culture reached an optical density (O.D. 600) of 0.500, a 1 µL aliquot was directly applied to a 1% agar-coated slide. The slide was left to air-dry for 5 minutes. These samples were promptly imaged using an Olympus IX-83 inverted microscope (manufactured by Olympus) equipped with a 60× phase-contrast objective.

## Data Availability

The mass spectrometry lipidomics data sets have been deposited to MetaboLights repository with the data set identifier MTBLS12808. The mass spectrometry proteomics data have been deposited to ProteomeXchange via the PRIDE partner repository with the data set identifier PXD066951.

## SUPPLEMENTAL MATERIAL

Figure S1 spectrum.00610-25-s0001.pdf GFP aggregation in response to isobutanol exposure.

Figure S2 spectrum.00610-25-s0002.pdf Membrane fatty acid composition of *Z. mobilis* and CFA Synthase.

Figure S3 spectrum.00610-25-s0003.pdf Anaerobic growth of *Z. mobilis* ZM4 in 7% ASGH diluted to 25%, 50%, and 100% (undiluted).

Table S1 spectrum.00610-25-s0004.xlsx Composition of 7% AFEX-pretreated switchgrass hydrolysate (ASGH).

Table S2 spectrum.00610-25-s0005.xlsx Doubling times of *Z. mobilis* grown in varying ASGH, ethanol, and isobutanol concentrations.

Table S3 spectrum.00610-25-s0006.xlsx Average Cell Area, Perimeter, and Circularity of *Z. mobilis* grown in ASGH, ethanol, and isobutanol concentrations.

Table S4 spectrum.00610-25-s0007.xlsx P-values* of the effects of ASGH, ethanol, isobutanol, and CFA synthase.

Table S5 spectrum.00610-25-s0008.xlsx Complete Proteomics dataset.

Table S6 spectrum.00610-25-s0009.xlsx Concentrations of End-Products of *Z. mobilis* Grown in 25%, 50%, and 100% Hydrolysate (7% ASGH).

ASM does not own the copyrights to Supplemental Material that may be linked to, or accessed through, an article. The authors have granted ASM a non-exclusive, world-wide license to publish the Supplemental Material files. Please contact the corresponding author directly for reuse.
