# Supplementary material for: Physiological and metabolic responses of Zymomonas mobilis to lignocellulosic hydrolysate
Source: Microbiol Spectr. 2025 Sep 17;13(10):e00610-25. doi: 10.1128/spectrum.00610-25 (PMC12502597; doi:10.1128/spectrum.00610-25)
Supplement: Figure S1 — GFP aggregation in response to isobutanol exposure. [file spectrum.00610-25-s0001.pdf]

Figure S1

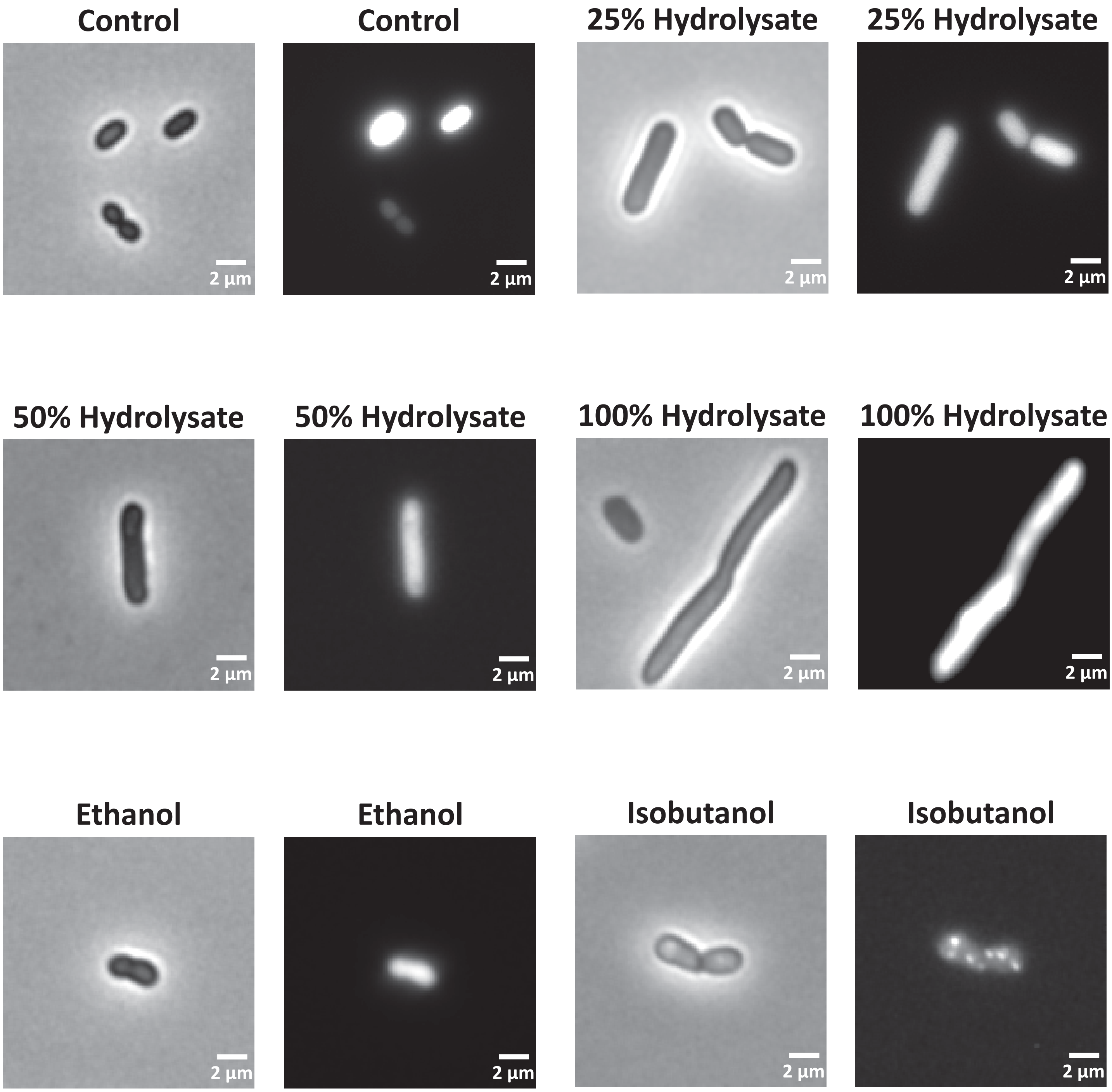

GFP aggregation in response to isobutanol exposure. A *Z. mobilis* strain engineered to express Green Fluorescent Protein (GFP) was grown anaerobically in minimal medium, ASGH diluted to 25%, 50%, and 100% (undiluted), or minimal medium supplemented with ethanol (0.80 M) or isobutanol (0.15 M). Fluorescence microscopy revealed that GFP aggregates formed exclusively in cultures exposed to isobutanol but were not observed during growth on hydrolysate or ethanol.
