# Supplementary material for: Physiological and metabolic responses of Zymomonas mobilis to lignocellulosic hydrolysate
Source: Microbiol Spectr. 2025 Sep 17;13(10):e00610-25. doi: 10.1128/spectrum.00610-25 (PMC12502597; doi:10.1128/spectrum.00610-25)
Supplement: Figure S2 — Membrane fatty acid composition of Z. mobilis and CFA Synthase. [file spectrum.00610-25-s0002.pdf]

Figure S2

Fatty Acid	WT	WT Hydrolysate			CFA	CFA *Z. mobilis* Hydrolysate			WT	WT
		25%	50%	100%	*Z. mobilis*	25%	50%	100%	Ethanol	Isobutanol
16:1	2.08	2.21	1.95	0.97	1.49	1.30	1.60	0.76	1.48	0.47
14:1	0.27	0.33	0.45	0.29	0.23	0.19	0.40	0.22	0.17	0.08
18:1	79.58	74.46	69.97	63.78	78.55	76.21	70.70	63.42	75.72	76.52
19:Cyclo	1.57	1.49	1.00	0.35	5.92	2.39	2.11	0.85	4.20	0.81
14:0	5.05	7.24	8.78	9.26	4.76	6.03	8.51	8.47	4.09	3.60
16:0	11.45	14.27	17.85	25.34	9.06	13.87	16.68	26.28	14.34	18.52

Membrane fatty acid composition of *Z. mobilis* and CFA Synthase Overexpressing *Z. mobilis* strain grown anaerobically in minimal media (control), ASGH diluted to 25%, 50%, and 100% (undiluted), or minimal media supplemented with ethanol (0.80 M) or isobutanol (0.15 M). The values indicate the mean weight percentages of fatty acids from three to four independent biological replicates, normalized to total membrane fatty acid content. Cell colors indicate the percent difference relative to the wild-type control, with blue denoting a decrease and red an increase, spanning a range from -16.2% to +14.8%. Fatty acid names indicate the number of carbon atoms and unsaturations; 19:Cyclo corresponds to cis-10,11-methyleneoctadecanoic acid.
