# Supplementary material for: Physiological and metabolic responses of Zymomonas mobilis to lignocellulosic hydrolysate
Source: Microbiol Spectr. 2025 Sep 17;13(10):e00610-25. doi: 10.1128/spectrum.00610-25 (PMC12502597; doi:10.1128/spectrum.00610-25)
Supplement: Figure S3 — Anaerobic growth of Z. mobilis ZM4 in 7% ASGH diluted to 25%, 50%, and 100% (undiluted). [file spectrum.00610-25-s0003.pdf]

Figure S3

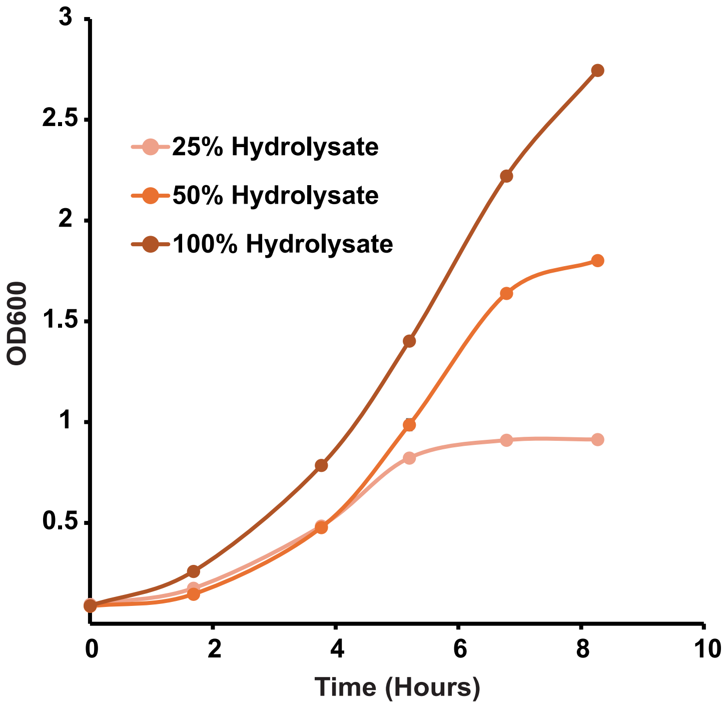

Anaerobic growth of *Z. mobilis* ZM4 in 7% ASGH diluted to 25%, 50%, and 100% (undiluted). Each data point represents the average of three biological replicates, with error bars indicating standard deviation.
